# Effects of yeast inoculation on community, metabolites, and flavors in ganjang, a traditional Korean fermented soy sauce

**DOI:** 10.1016/j.fochx.2026.104200

**Published:** 2026-07-13

**Authors:** Dong Min Han, Ju Hye Baek, Dae Gyu Choi, Jae Kyeong Lee, Byung Hee Chun, Che Ok Jeon

**Affiliations:** a Department of Life Science, Chung-Ang University, Seoul 06974, Republic of Korea; b Department of Microbiology, Pukyong National University, Busan 48513, Republic of Korea

**Keywords:** Ganjang fermentation, Soy sauce, Yeast starter, Volatile organic compounds, Microbial community, Metabolites

## Abstract

We investigated the effects of defined yeast starters on ganjang fermentation using batches inoculated with *Debaryomyces hansenii*, *Wickerhamomyces anomalus*, and/or *Wickerhamiella versatilis* in combination with *Tetragenococcus halophilus*. Absolute quantification showed that *T. halophilus* dominated the bacterial community and was associated with early acidification, whereas yeasts persisted at low abundance and became readily detectable only after doenjang-meju removal. Yeast inoculation had minimal effects on pH, microbial community structure, and primary metabolite profiles. In contrast, volatile organic compound (VOC) profiles clearly differentiated yeast-inoculated batches in a fermentation stage-dependent manner; *Wi. versatilis* and *D. hansenii* were associated with more diverse VOC profiles at 60 and 240 days, respectively. Notably, *Wi. versatilis* was associated with distinct VOC profiles despite its low abundance. These findings suggest the stage-specific roles of defined yeasts and support targeted yeast selection as a strategy to modulate VOC profiles in high-salinity ganjang fermentation.

## Highlights

• Absolute quantification revealed yeast effects masked by relative community analysis.
• Yeast inoculation minimally affected metabolites but strongly altered volatile profiles.
• *Debaryomyces hansenii* and *Wickerhamiella versatilis* enhanced flavor complexity.
• Low-abundance yeasts exerted significant impacts on ganjang flavor.
• Targeted yeast starters offer a strategy to modulate ganjang aroma.

## 1 Introduction

Ganjang, a traditional Korean fermented soybean sauce, is produced through long-term brine fermentation of fermented soybean bricks (doenjang-meju) and develops its characteristic sensory-related VOC profiles through complex, dynamic microbial successions (Chun et al., 2021; Han et al., 2020; Jung et al., 2015). Increasing evidence indicates that ganjang quality is primarily shaped by microbial metabolism, particularly via the production and transformation of metabolites and volatile organic compounds (VOCs) during fermentation (Byeon et al., 2023). While halophilic lactic acid bacteria such as *Tetragenococcus* (*T.*) *halophilus*, one of the key bacterial species in ganjang fermentation, play central roles in acidification and fermentation stability (Chun et al., 2019, Chun et al., 2021; Han et al., 2020), yeasts are considered important contributors to VOC formation by converting sugars, amino acids, and lipids into diverse aroma-active compounds, including esters, alcohols, aldehydes, and phenolic compounds (Wang et al., 2024; Yoo et al., 2025).

In soy-based fermentations, yeasts such as *Zygosaccharomyces rouxii*, *Wickerhamiella* (*Wi*.) *versatilis* (previously *Candida versatilis*), and *Pichia guilliermondii* have been identified as key contributors to VOC formation (Ma et al., 2025; Mizuno et al., 2025; Wang et al., 2022). In addition, *Debaryomyces* and *Wickerhamomyces* species have been repeatedly associated with VOC profile development through amino acid and lipid metabolism in ganjang and other fermented foods, highlighting their multifunctional roles beyond carbohydrate fermentation (Chun et al., 2021; Jeong et al., 2022; Lu et al., 2021; Riahi et al., 2007; Zhang et al., 2021). Despite their recognized importance, most previous studies have focused primarily on relative microbial community composition or bulk metabolite profiles, approaches that may obscure true microbial dynamics and species-specific functional contributions during batch fermentation (Han et al., 2020; Song et al., 2021). Moreover, previous studies of ganjang fermentation have largely relied on spontaneous microbial assemblages, further limiting the ability to resolve the specific roles of individual yeasts in shaping fermentation outcomes.

Ganjang fermentation is a batch process in which both microbial community composition and total population size change markedly over time (Chun et al., 2021). Although relative abundance-based analysis is the most commonly used approach for microbial community studies, it can misrepresent actual microbial dynamics when total population sizes shift substantially during fermentation (Wang, Li, et al., 2025). Thus, taxa with high relative abundance are not necessarily numerically dominant or metabolically important at a given stage. Therefore, absolute abundance analysis is required to accurately assess functional importance and to evaluate the specific contributions of individual microorganisms during ganjang fermentation. In this context, “absolute abundance” refers to the estimated abundance of microbial gene copies or cells per unit volume (e.g., per mL of ganjang), rather than their relative proportions within the community.

In ganjang fermentation, *Debaryomyces* (*D.*) *hansenii*, *Wickerhamomyces* (*Wo.*) *anomalus*, and *Wi. versatilis* are consistently detected as dominant or recurrent yeast species (Chun et al., 2021; Han et al., 2020; Song et al., 2021). However, their specific functional roles in shaping microbial succession, metabolite production, and VOC formation during different fermentation stages remain unclear. Therefore, the objective of this study was to determine the contributions of these representative yeasts during ganjang fermentation using controlled inoculation experiments in combination with *T. halophilus*. We integrated absolute microbial quantification with metabolite and VOC profiling to distinguish yeast-specific effects from background microbial dynamics. This study will provide new insights into the functional roles of key ganjang-associated yeasts and provide a possible framework for the rational selection of yeast starters to modulate aroma-associated VOC profiles in high-salinity liquid fermentations.

## 2 Materials and methods

### 2.1 Ganjang preparation and sampling

An overview of the experimental design, including inoculation sets and sampling workflow, is provided in Fig. S1. Briefly, five sets of ganjang batches were prepared in triplicate following previously described methods with minor modifications (Chun et al., 2021; Han et al., 2020), with the following inoculation sets: T (*T. halophilus* MJ4), TD (*T. halophilus* + *D. hansenii* KD2), TW (*T. halophilus* + *Wo. anomalus* A11–1-30), TC (*T. halophilus* + *Wi. versatilis* C0–1-P4), and TDWC (*T. halophilus* + *D. hansenii* + *Wo. anomalus* + *Wi. versatilis*); all strains were isolated in our laboratory from traditional Korean fermented foods. The inoculum level (approximately 10^6^ cells/mL for each strain) was selected based on microbial population sizes observed during active ganjang fermentation in our previous study (Chun et al., 2021). All strains were inoculated at the beginning of fermentation to provide identical initial conditions among treatments and to evaluate the effects of yeast starters throughout the entire fermentation process. For inoculum preparation, *T. halophilus* was cultivated in tryptic soy broth (MBcell, South Korea) supplemented with 5% (*w*/*v*) NaCl, while yeast strains were cultured in yeast extract-peptone-dextrose (YPD; MBcell) broth containing 5% (w/v) NaCl. After culture, cells were harvested by centrifugation and resuspended prior to inoculation. For ganjang preparation, solar salts (produced in Shinan, Korea) were dissolved in tap water to a final concentration of approximately 16% (w/v) and left overnight to allow insoluble impurities to settle. Four doenjang-meju bricks (approximately 5 kg total) were then immersed in 20 L of the clarified salt solution in Korean porcelain fermentation pots. The doenjang-meju bricks had been previously prepared using *Aspergillus* (*A.*) *oryzae* MA1 (10^3^ spores/g) and *Bacillus* (*B.*) *velezensis* MB1 (10^6^ cells/g), as described previously (Han et al., 2023).

Fermentation proceeded at room temperature (approximately 25–30 °C) for 60 days. The doenjang-meju bricks were then removed from the liquid phase, and the resulting ganjang was aged for an additional 180 days under the same conditions. Samples collected at 0, 5, 20, 40, 60, 70, 100, 150, and 240 days were measured for their pH values. The samples were centrifuged (18,894 ×*g*, 10 min, 4 °C) to collect microbial pellets and supernatants. Microbial pellets pooled from equal volumes of triplicate batches were stored at −80 °C for microbial analysis, while supernatants were stored separately at −80 °C for metabolite and flavor compound analyses.

### 2.2 Bacterial and fungal community analysis

For the relative and absolute bacterial and fungal community analyses, known quantities of *Neobacillus terrae* C11 (GenBank accession no. JAAOEO000000000; 5 × 10^7^ cells) and *Ogataea parapolymorpha* DL-1 (GenBank accession no. AEOI00000000; 5 × 10^6^ cells), which are not naturally present in ganjang, were added to each microbial pellet sample derived from 10 mL of ganjang prior to DNA extraction as internal standards. The cell numbers of *N. terrae* and *O. parapolymorpha* were determined by colony-forming unit (CFU) counts on R2A (MBcell) and YPD agar media, respectively. Genomic DNA was extracted from the microbial pellets using the FastDNA™ Spin Kit for Soil (MP Biomedicals, USA) according to the manufacturer's protocol. The V3-V4 regions of bacterial 16S rRNA genes and the ITS2 regions of fungal rRNA genes were amplified as described previously (Han et al., 2020). The PCR amplicons were pooled and sequenced on the Illumina MiSeq paired-end platform (2 × 300 bp; Roche, USA) at Macrogen (South Korea). Raw reads underwent processing in QIIME2 (version 2023.05; Bolyen et al., 2019), including demultiplexing by barcode, trimming adapter and barcode sequences with Cutadapt, quality filtering (Q ≥ 25) and denoising, paired-end merging with the DADA2 plugin, and chimera removal. The 16S rRNA gene reads of *N. terrae* and ITS region reads of *O. parapolymorpha* were excluded from the datasets before downstream analysis. Singleton amplicon sequence variants (ASVs) were removed, and taxonomic classification at the genus level applied the classify-sklearn method with SILVA (bacteria) and UNITE (fungi) databases, within QIIME2.

Absolute abundances of bacterial 16S rRNA genes and fungal ITS genes were estimated at the genus level by normalizing sequencing read counts of each taxon to those of the corresponding internal standards added to each sample. The calculations incorporated the known cell numbers and marker gene copy numbers of the internal standards (one 16S rRNA gene copy per *N. terrae* cell and five ITS copies per *O. parapolymorpha* cell). Accordingly, gene copy abundances for each genus were estimated from the ratio of sample reads to internal-standard reads and expressed as estimated gene copies per mL of ganjang. Relative community compositions were calculated using sequencing reads after excluding those derived from the internal standards.

### 2.3 Analysis of metabolic compounds

The concentrations of organic compounds, including free sugars, organic acids, and amino acids, in ganjang batches were analyzed using ^1^H NMR spectroscopy, as previously described (Baek et al., 2023). Briefly, 0.5 mL of each ganjang sample was mixed with an equal volume of 99.9% D_2_O (Sigma-Aldrich, USA) containing 10 mM sodium 2,2-dimethyl-2-silapentane-5-sulfonate (DSS; Sigma-Aldrich). The mixtures were centrifuged at 4 °C at 12,300 ×*g* for 10 min, and 700 μL of the resulting supernatant was transferred into NMR tubes. ^1^H NMR spectra were acquired using a Varian Inova 600-MHz spectrometer (Varian, USA) and metabolite concentrations were quantified using the Chenomx NMR Suite software (version 6.1; Chenomx, Canada).

The concentrations of flavonoid aglycones in ganjang batches were analyzed using a 1290 Infinity ultra-high-performance liquid chromatograph equipped with a 6550 iFunnel Q-TOF mass spectrometer (Agilent Technologies, USA), as previously described (Baek et al., 2024) with minor modifications. Briefly, each ganjang sample was mixed with an equal volume of extraction solution (methanol:acetonitrile = 1:1) containing 20 μM reserpine as an internal standard and centrifuged at 4 °C and 12,300 ×*g* for 10 min. The supernatants were filtered through a 0.2-μm polyvinylidene fluoride membrane filter (BioFACT, Korea) and injected into an Eclipse Plus C18 column (2.1 mm × 100 mm; Agilent Technologies). Water (A) and acetonitrile (B), both containing 0.1% formic acid, were used as mobile phases at a flow rate of 0.3 mL/min under the following gradient conditions: 0 min, 5% (*v*/v) B; 1 min, 5% B; 18 min, 95% B; 19 min, 95% B; 20 min, 5% B. Mass spectrometry was conducted under the following conditions: polarity, positive; gas temperature, 250 °C; nebulizer, 35 psi; capillary, (+) 4000 V; MS scan range, 30–1050 *m*/*z*; MS/MS scan range, 20–1050 m/z; and collision-induced dissociation energy, 20 eV. Flavonoid aglycones in ganjang batches were identified and quantified using authentic reference standards purchased from TCI (Japan) and Sigma-Aldrich.

### 2.4 Analysis of VOCs

VOCs in the ganjang batches were analyzed using headspace solid-phase microextraction (HS-SPME) coupled with gas chromatography–mass spectrometry (GC/MS), following a previously described method (Han et al., 2021; 2023). Briefly, 1 mL of each ganjang sample was transferred into a 20-mL SPME vial sealed with a silicone/Teflon septum (Supelco, USA), and methyl cinnamate (Sigma-Aldrich) in methanol was added as an internal standard at a final concentration of 10 μg/mL. HS-SPME was conducted at 50 °C for 30 min using an Agilent 120 PAL autosampler equipped with a 2-cm divinylbenzene/carboxen/polydimethylsiloxane (DVB-CAR-PDMS) fiber (Supelco, USA). Thermal desorption of VOCs adsorbed on the fiber occurred in the GC injection port at 250 °C for 2 min and analyzed in splitless mode using an HP-GC/MS system (7820 A/5977E MSD, Agilent Technologies) equipped with a DB-Wax column (50 m × 200 μm × 0.2 μm; Agilent Technologies). The GC oven program was as follows: 40 °C for 5 min; ramp to 150 °C at 5 °C/min; ramp to 200 °C at 7 °C/min; hold at 200 °C for 10 min. Helium served as the carrier gas at a constant flow rate of 1.5 mL/min. Peak areas of VOCs were normalized to the peak area of methyl cinnamate in each sample. Because absolute quantification was not performed, the concentrations of VOCs were expressed as relative percentages, calculated by assuming proportionality between peak area and concentration and normalizing to the total peak area of the sample with the highest overall signal (the 40-day ganjang sample in the TDWC batch). Accordingly, VOC data were treated as semi-quantitative and used for comparative analysis rather than absolute quantification of aroma intensity. VOCs were tentatively identified based on comparisons of their mass spectra with those in the NIST mass spectral library. Because authentic reference standards, retention index (RI) confirmation, GC-O analysis, and MS/MS validation were not available for all detected compounds, VOCs reported in this study should be regarded as tentatively identified compounds.

### 2.5 Statistical analyses

Metabolite and VOC profiles of ganjang batches prepared with different starter combinations at 60 and 240 days were analyzed using principal component analysis (PCA) implemented with the *prcomp* function in R. Differences in metabolite and VOC profiles among batches were further evaluated using permutational multivariate analysis of variance (PERMANOVA; 999 permutations) with the *pairwise.adonis* function in the vegan package (v2.6–4) in R (https://cran.r-project.org/web/packages/vegan/). In addition, permutational analysis of multivariate dispersions (PERMDISP) was conducted using *betadisper*, followed by permutation testing with *permutest*, to assess the effect of group dispersion. To identify metabolites and VOCs that significantly discriminated among batches, analysis of variance (ANOVA) was performed using MetaboAnalyst (https://www.metaboanalyst.ca/MetaboAnalyst) (Pang et al., 2024). Multiple-comparison correction was performed in MetaboAnalyst using the Benjamini-Hochberg false discovery rate (FDR) method (Benjamini & Hochberg, 1995). Metabolites and VOCs with FDR-adjusted *p*-values <0.05 were considered statistically significant. Reported aroma-associated characteristics of VOCs showing statistically significant differences among ganjang batches at 60 and 240 days were annotated based on information from FooDB (https://foodb.ca/) and visualized using donut charts.

## 3 Results

### 3.1 Preparation of ganjang batches with different yeast inoculations

To investigate the fermentation characteristics of yeasts during ganjang fermentation, ganjang batches inoculated with different yeasts were prepared in triplicate. To ensure consistency across all batches, doenjang-meju bricks fermented in this study with *A. oryzae* and *B. velezensis*, the predominant and functionally important microorganisms in doenjang-meju fermentation, were used as the primary raw material instead of naturally fermented doenjang-meju bricks (Han et al., 2023; Kim et al., 2022). In addition, *T. halophilus*, a halophilic lactic acid bacterium commonly associated with ganjang fermentation (Chun et al., 2021; Han et al., 2020), was inoculated as a background starter in all ganjang batches. All other materials used for ganjang preparation were identical across batches, except for the yeast inoculation combinations.

**Fig. 1:**
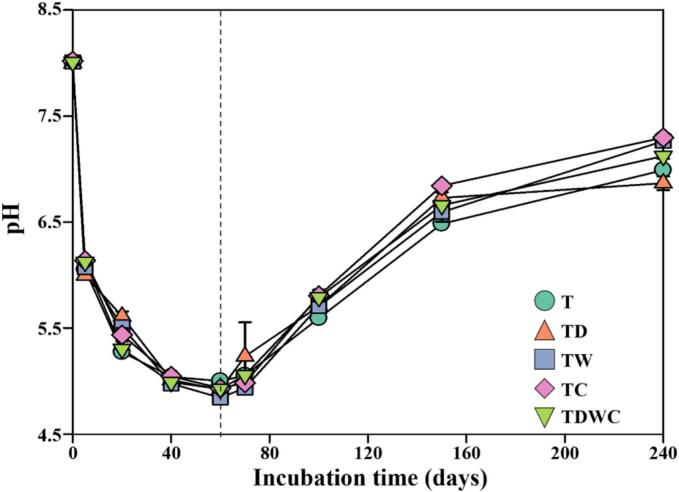
pH profiles of ganjang batches inoculated with *Tetragenococcus halophilus* (T), *T. halophilus* and *Debaryomyces hansenii* (TD), *T. halophilus* and *Wickerhamomyces anomalus* (TW), *T. halophilus* and *Wickerhamiella versatilis* (TC), and *T. halophilus*, *D. hansenii*, *Wo. anomalus*, and *Wi. versatilis* (TDWC) during fermentation. The dotted line indicates the day on which the doenjang-meju bricks were removed from the ganjang solution (day 60). Data are presented as mean values ± standard errors from triplicate samples.

Reflecting these standardized conditions, the initial pH values of all ganjang batches were approximately 8.0, regardless of yeast inoculation (Fig. 1). The pH rapidly decreased during the early fermentation period and gradually declined to approximately 4.8–5.0, showing highly similar profiles across batches until the doenjang-meju bricks were removed from the ganjang solution at 60 days. After removal of the doenjang-meju bricks, the pH values continuously increased in all batches, reaching slightly different final levels (6.7–7.3) by the end of fermentation (240 days).

### 3.2 Bacterial and fungal community changes in ganjang batches with different yeast inoculations during fermentation

To more accurately identify key microbial contributors, both relative and absolute analyses of bacterial and fungal communities were performed. For absolute quantification, known amounts of the bacterium *N. terrae* and the yeast *O. parapolymorpha*, which are absent from ganjang, were added in equal amounts to all samples prior to DNA extraction as internal standards. Sequencing reads were then normalized to these reference strains, enabling visualization of microbial communities in both relative and absolute terms.

**Fig. 2:**
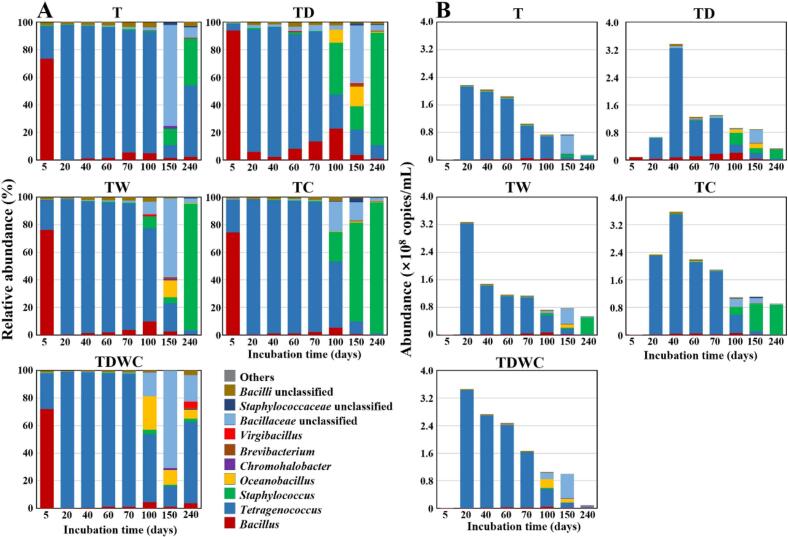
Relative (A) and estimated absolute (B) bacterial communities at the genus level during fermentation in ganjang batches inoculated with *Tetragenococcus halophilus* (T), *T. halophilus* and *Debaryomyces hansenii* (TD), *T. halophilus* and *Wickerhamomyces anomalus* (TW), *T. halophilus* and *Wickerhamiella versatilis* (TC), and *T. halophilus*, *D. hansenii*, *Wo. anomalus*, and *Wi. versatilis* (TDWC). “Others” represent all bacterial genera with relative abundances below 1.0% across all samples.

Relative bacterial community analysis showed that *Bacillus*, the bacterial starter used in doenjang-meju production, predominated at day 5 in all batches (Fig. 2A). However, it declined sharply and nearly disappeared by day 20. *Tetragenococcus*, the common starter inoculated into all batches, rapidly became dominant and remained the major taxon even after doenjang-meju removal (60 days). At 100 days, *Oceanobacillus*, unclassified *Bacillaceae*, and *Staphylococcus* increased in relative abundance, with unclassified *Bacillaceae* and *Staphylococcus* dominating certain batches at 150–240 days. *Tetragenococcus* remained dominant, whereas *Bacillus* was mainly detected at early stages and unclassified *Bacillaceae/Staphylococcus* increased at later stages.

Absolute analysis, however, revealed a markedly different pattern (Fig. 2B). *Bacillus*, which appeared dominant on day 5 in the relative analysis, showed extremely low estimated absolute gene copy abundance despite high relative abundance. Total bacterial abundance peaked at days 20–40 across all batches, with *Tetragenococcus* clearly dominating. Although *Oceanobacillus*, unclassified *Bacillaceae*, and *Staphylococcus* appeared at day 100, their absolute abundances remained far lower than *Tetragenococcus*.

**Fig. 3:**
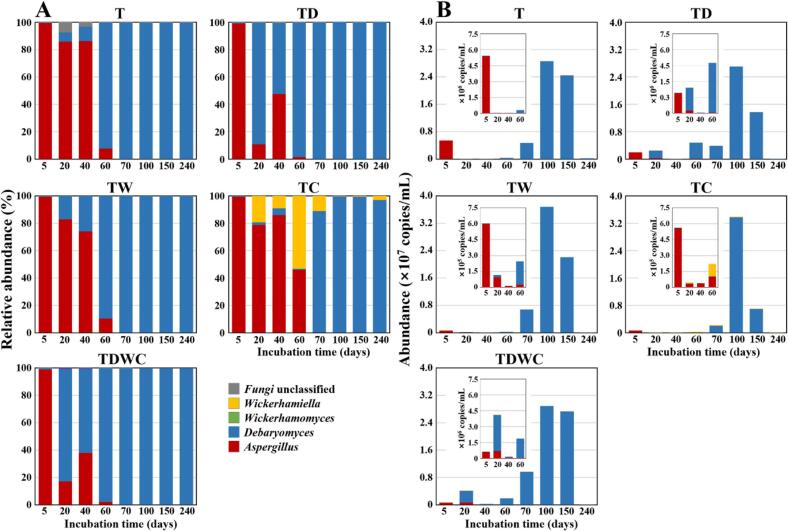
Relative (A) and estimated absolute (B) fungal community compositions at the genus level during fermentation in ganjang batches inoculated with *Tetragenococcus halophilus* (T), *T. halophilus* and *Debaryomyces hansenii* (TD), *T. halophilus* and *Wickerhamomyces anomalus* (TW), *T. halophilus* and *Wi. versatilis* (TC), and *T. halophilus*, *D. hansenii*, *Wo. anomalus*, and *Wi. versatilis* (TDWC). Insets in panel B show the absolute abundances of fungal taxa with a magnified scale for the period before meju brick removal (0–60 days).

Relative fungal community analysis showed that *Aspergillus*, the doenjang-meju starter, dominated early in all batches and remained predominant until doenjang-meju removal on day 60 (Fig. 3A). In batches inoculated with *D. hansenii* (TD and TDWC), *Debaryomyces* became dominant after day 5. In the TC batch inoculated with *Wi. versatilis*, *Wickerhamiella* increased markedly after day 5, but it was nearly absent in the TDWC batch where multiple fungal starters were co-inoculated. *Wo. anomalus*, despite being inoculated into the TW and TDWC batches, remained almost undetectable. After day 60, *Debaryomyces* became the predominant fungal taxon across all batches, with *Wi. versatilis* still detectable at low levels only in the TC batch.

Absolute fungal community analysis revealed substantial discrepancies compared with the relative results (Fig. 3B). Although *Aspergillus* appeared highly abundant before day 60 in the relative analysis, its absolute abundance during this period was extremely low, indicating that, like *Bacillus*, it originated only from the doenjang-meju bricks and did not grow in the ganjang environment, contributing minimally to ganjang fermentation. A similar pattern was observed for the inoculated yeasts, including *Debaryomyces*: despite appearing dominant in relative terms in the TD and TDWC batches, their absolute abundances remained very low until day 60. Following the removal of the doenjang-meju bricks, total fungal abundance increased sharply, peaked around day 100, and then gradually declined, becoming nearly undetectable by day 240.

### 3.3 Metabolite changes in ganjang batches with different yeast inoculations during fermentation

**Fig. 4:**
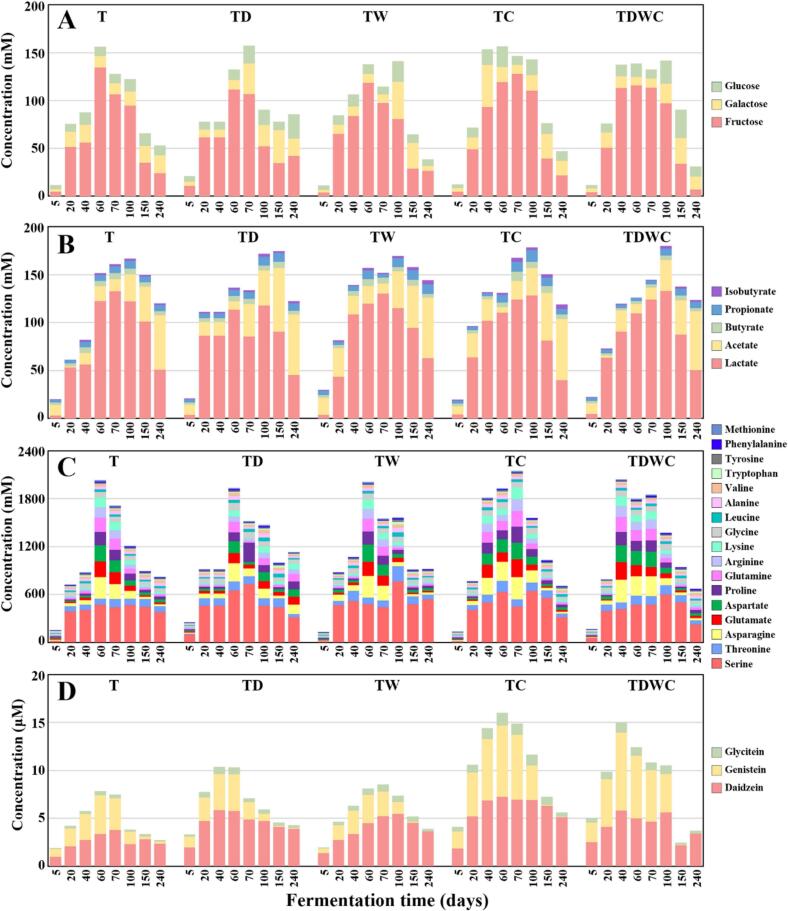
Profiles of free sugars (A), organic acids (B), amino acids (C), and flavonoid aglycones (D) during fermentation in ganjang batches inoculated with *Tetragenococcus halophilus* (T), *T. halophilus* and *Debaryomyces hansenii* (TD), *T. halophilus* and *Wickerhamomyces anomalus* (TW), *T. halophilus* and *Wickerhamiella versatilis* (TC), and *T. halophilus*, *D. hansenii*, *Wo. anomalus*, and *Wi. versatilis* (TDWC).

Major metabolites, including free sugars, organic acids, amino acids, and flavonoid aglycones, were profiled throughout fermentation, and their overall patterns were highly similar across all ganjang batches regardless of the yeast inoculation (Fig. 4). Excluding organic acids, the concentrations of free sugars, amino acids, and flavonoid aglycones gradually increased during the early fermentation period and then decreased after the removal of the doenjang-meju bricks. This trend is consistent with previous findings (Han et al., 2020). These metabolites likely accumulated early because they were progressively released from the doenjang-meju bricks into the ganjang solution; once the doenjang-meju bricks were removed, their input ceased, and microbial utilization resulted in a steady decline.

In contrast, organic acids accumulated continuously throughout fermentation (Fig. 4B). They were produced through microbial fermentation of free sugars before and after doenjang-meju removal, resulting in sustained increases that peaked around day 100, after which levels gradually decreased. Fructose, galactose, and glucose, particularly fructose, were the major free sugars detected in all batches (Fig. 4A), consistent with previous analyses of sugars present in doenjang-meju (Han et al., 2023; Kim et al., 2022). Lactate and acetate were the dominant organic acids. Lactate increased sharply until approximately day 100 and then declined rapidly, whereas acetate increased steadily throughout fermentation and became the most abundant organic acid at the final stage. Butyrate, propionate, and isobutyrate were also detected, but their levels remained relatively constant. Amino acids, key contributors to taste in fermented foods, increased during early fermentation but declined noticeably after doenjang-meju removal (Fig. 4C). Serine was the most abundant amino acid across all batches, and interestingly, unlike other amino acids, its concentration remained relatively constant even after the doenjang-meju bricks were removed.

**Fig. 5:**
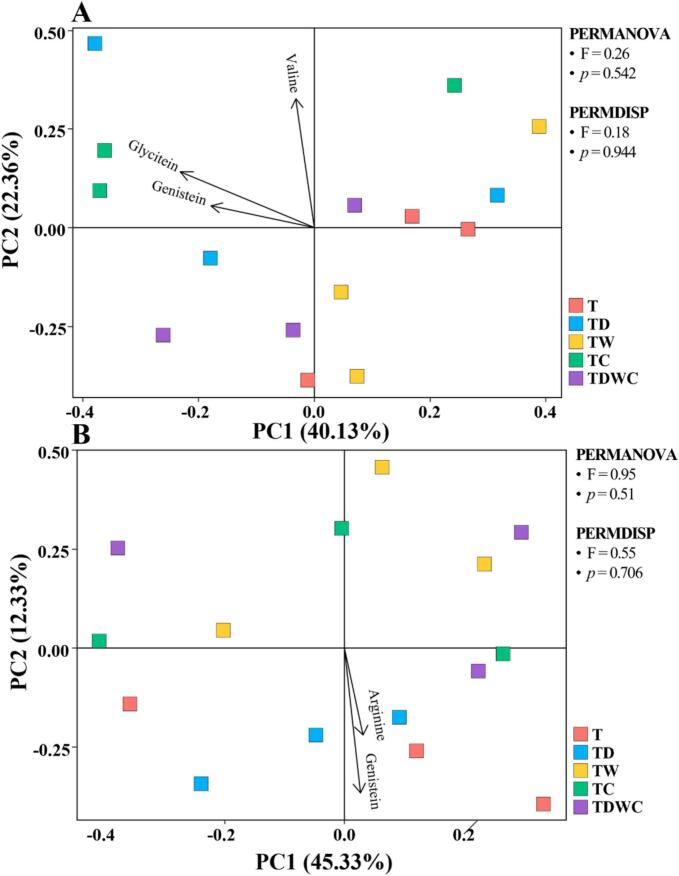
Principal component biplots of metabolite profiles, including free sugars, organic acids, amino acids, and flavonoid aglycones, in ganjang batches inoculated with *Tetragenococcus halophilus* (T), *T. halophilus* + *Debaryomyces hansenii* (TD), *T. halophilus* + *Wickerhamomyces anomalus* (TW), *T. halophilus* + *Wickerhamiella versatilis* (TC), and *T. halophilus* + *D. hansenii* + *Wo. anomalus* + *Wi. versatilis* (TDWC) at 60 days (A) and 240 days (B). Differences in metabolite profiles among batches were assessed using PERMANOVA, and dispersion was evaluated using PERMDISP. Only metabolites showing significant differences among batches (Benjamini-Hochberg FDR-adjusted *p* < 0.05) are shown as vectors, with arrow lengths proportional to their contributions to overall variation.

Daidzein, genistein, and glycitein, particularly daidzein and genistein, were the major flavonoid aglycones detected, consistent with previous findings in doenjang (Han et al., 2023; Kim et al., 2022) (Fig. 4D). Daidzein levels remained relatively constant throughout fermentation, whereas genistein increased rapidly and then decreased sharply after doenjang-meju removal. Flavonoid aglycone levels also showed modest batch-specific differences: concentrations were slightly higher in the TC and TDWC batches and lowest in the T batch. PCA based on all detected metabolites, including free sugars, organic acids, amino acids, and flavonoid aglycones, in ganjang batches at 60 and 240 days showed that overall metabolite profiles were not clearly separated by different yeast inoculation combinations (Fig. 5). PERMANOVA, which tests differences in multivariate centroid locations among groups, showed no significant effect of yeast inoculation on metabolite profiles (60 days: F = 0.26, *p* = 0.542; 240 days: F = 0.95, *p* = 0.510). PERMDISP, which tests differences in within-group dispersion, likewise indicated no significant differences among batches (60 days: F = 0.18, *p* = 0.944; 240 days: F = 0.55, *p* = 0.706). Together, these results further support the absence of significant effects of yeast inoculation on overall metabolite composition.

### 3.4 Changes in VOC profiles of ganjang batches with different yeast inoculations during fermentation

**Fig. 6:**
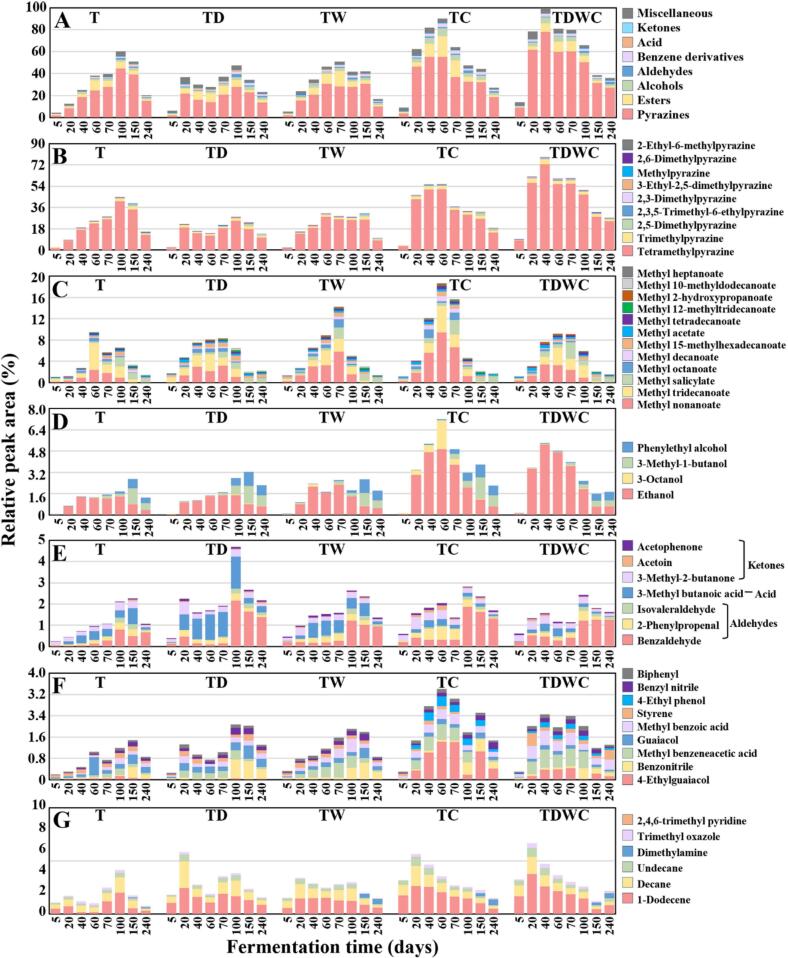
Profiles of volatile organic compounds (VOCs) during fermentation in ganjang batches inoculated with *Tetragenococcus halophilus* (T), *T. halophilus* and *Debaryomyces hansenii* (TD), *T. halophilus* and *Wickerhamomyces anomalus* (TW), *T. halophilus* and *Wickerhamiella versatilis* (TC), and *T. halophilus*, *D. hansenii*, *Wo. anomalus*, and *Wi. versatilis* (TDWC). A, total VOCs; B, pyrazines; C, esters; D, alcohols; E, aldehydes, acids, and ketones; F, benzene derivatives; and G, miscellaneous compounds. VOCs were tentatively identified based on GC/MS spectral matching. Relative VOC abundances were expressed as percentages, calculated based on the total peak area of the sample with the highest overall signal (the 40-day ganjang sample in the TDWC batch in panel A).

All VOCs discussed below were tentatively identified based on GC/MS spectral matching unless otherwise stated. Analysis of VOCs revealed a diverse array of chemical classes, including pyrazines, esters, alcohols, aldehydes, organic acids, ketones, and benzene derivatives, across all ganjang samples (Table S1; Fig. 6). Among these, pyrazines were the most abundant class, with tetramethylpyrazine representing more than 50% of the total VOCs, making it the predominant VOC detected throughout fermentation. In addition to pyrazines, a range of esters (e.g., methyl nonanoate, methyl tridecanoate, methyl salicylate), alcohols (e.g., ethanol, 3-octanol, 3-methyl-1-butanol), aldehydes (e.g., benzaldehyde, 2-phenylpropenal), acids (e.g., 3-methylbutanoic acid), and benzene derivatives (e.g., 4-ethylguaiacol, benzonitrile, methyl benzeneacetic acid, guaiacol) were also identified at varying abundances.

Unlike microbial community profiles and metabolite patterns, which remained highly similar across batches, VOC profiles differed substantially depending on the yeast starter. Notably, the TC and TDWC batches inoculated with *Wi. versatilis* exhibited higher overall relative VOC abundances compared with the other batches.

VOC dynamics also varied considerably depending on fermentation stage. Many esters, including methyl nonanoate, methyl tridecanoate, methyl decanoate, and methyl octanoate, were most abundant during the mid-fermentation period, whereas methyl salicylate accumulated primarily during late fermentation. Ethanol increased continuously until the doenjang-meju bricks were removed, while 3-methyl-1-butanol and phenylethyl alcohol increased mainly after doenjang-meju removal. Aldehydes showed similar stage-specific shifts across batches, with benzaldehyde exhibiting a pronounced increase at late fermentation stages. Several benzene derivatives, such as methyl benzeneacetic acid, guaiacol, and methyl benzoic acid, remained abundant throughout the entire fermentation period, whereas others such as 4-ethylguaiacol and benzonitrile were detected only in specific batches or at particular stages. Among acids, 3-methylbutanoic acid was the only one consistently detected and was most abundant during mid-fermentation.

**Fig. 7:**
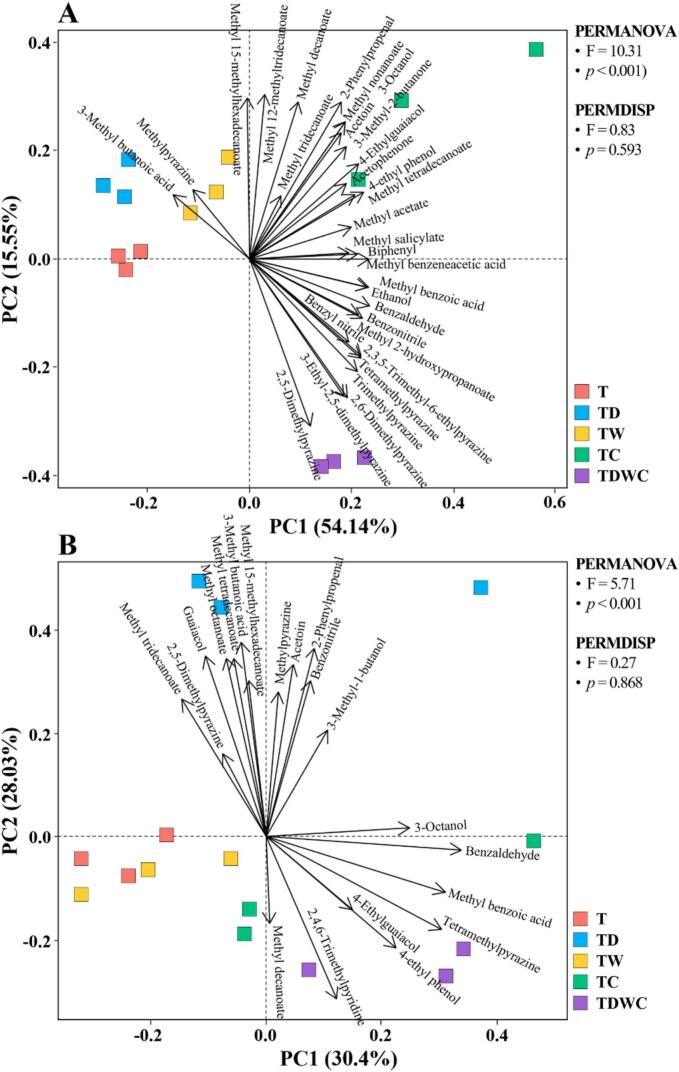
Principal component biplots of volatile organic compounds (VOCs) in ganjang batches inoculated with *Tetragenococcus halophilus* (T), *T. halophilus* + *Debaryomyces hansenii* (TD), *T. halophilus* + *Wickerhamomyces anomalus* (TW), *T. halophilus* + *Wickerhamiella versatilis* (TC), and *T. halophilus* + *D. hansenii* + *Wo. anomalus* + *Wi. versatilis* (TDWC) at 60 days (A) and 240 days (B). VOCs were tentatively identified based on GC/MS spectral matching. Differences in VOC profiles among batches were assessed using PERMANOVA, and dispersion was evaluated using PERMDISP. Only VOCs showing significant differences among batches (Benjamini-Hochberg FDR-adjusted *p* < 0.05) are shown as vectors, with arrow lengths proportional to their contributions to multivariate variation; these VOCs represent compounds potentially associated with sensory differentiation among treatments.

PCA based on the VOCs identified in the ganjang batches at 60 and 240 days demonstrated that overall metabolite profiles were clearly separated by different yeast inoculation combinations (Fig. 7; Table S2), in contrast to the largely similar metabolite profiles observed in Fig. 5. PERMANOVA confirmed significant differences in VOC composition among inoculation groups (60 days: F = 10.31, *p* < 0.001; 240 days: F = 5.71, *p* < 0.001), whereas PERMDISP indicated no significant differences in within-group dispersion (60 days: F = 0.83, *p* = 0.593; 240 days: F = 0.27, *p* = 0.868). Together, these results demonstrate that yeast inoculation significantly affected overall VOC profiles. Notably, the PCA biplot revealed a more pronounced separation among batches at 60 days than at 240 days. At 60 days, TC- and TDWC- inoculated batches, particularly the TC-inoculated batch, exhibited more diverse and higher relative VOC abundance profiles, whereas at 240 days, higher VOC diversity and relative abundance were observed in the TD-, TDWC-, and TC-inoculated batches, particularly in the TD-inoculated batch. These results indicate that even yeasts present at low abundance may contribute to shifts in VOC composition.

**Fig. 8:**
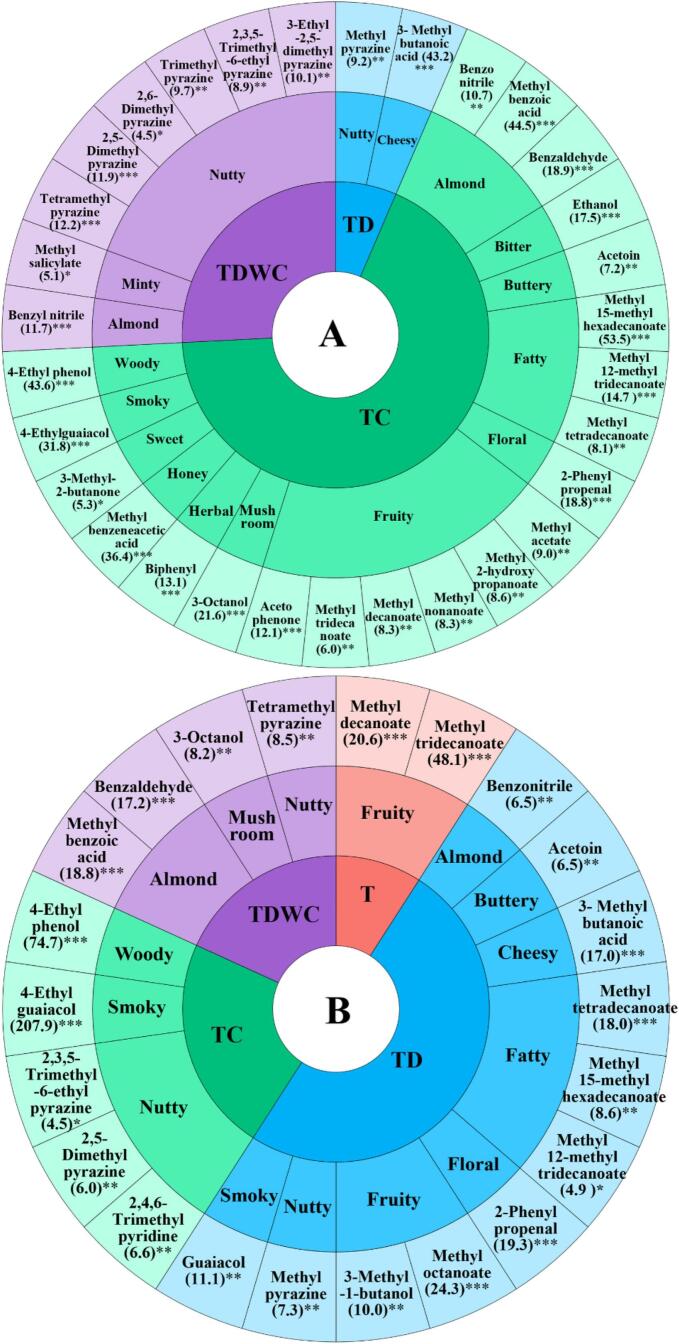
Flavor characteristics of volatile organic compounds (VOCs) showing statistically significant differences among ganjang batches inoculated with *Tetragenococcus halophilus* (T), *T. halophilus* + *Debaryomyces hansenii* (TD), *T. halophilus* + *Wickerhamomyces anomalus* (TW), *T. halophilus* + *Wickerhamiella versatilis* (TC), and *T. halophilus* + *D. hansenii* + *Wo. anomalus* + *Wi. versatilis* (TDWC) at 60 days (A) and 240 days (B). VOCs were tentatively identified based on GC/MS spectral matching. Only VOCs showing significant differences among batches (Benjamini-Hochberg FDR-adjusted *p* < 0.05) are presented. Flavor annotations (e.g., smoky, woody, fruity, nutty, cheesy, floral) were assigned based on FooDB. Numbers in parentheses indicate F values from ANOVA, reflecting their relative discriminant power. These compounds represent the principal discriminant VOCs potentially associated with yeast-dependent sensory differentiation at each fermentation stage. Statistical significance is denoted as *p* < 0.05 (*), *p* < 0.01 (**), and *p* < 0.001 (***).

Analysis of VOCs associated with putative aroma-related characteristics showing statistically significant differences among ganjang batches at 60 and 240 days further supported this observation (Fig. 8). At 60 days, TC-inoculated batches exhibited significantly higher relative abundances of several VOCs, including 4-ethylguaiacol (smoky-associated), 4-ethylphenol (woody-associated), methyl benzeneacetic acid (honey-like-associated), methyl benzoic acid (almond-like-associated), 3-octanol (mushroom-like-associated), methyl 15-methylhexadecanoate and methyl 12-methyltridecanoate (fatty-associated), as well as acetophenone and methyl acetate (fruity-associated) (Fig. 8A). In contrast, TDWC-inoculated batches were characterized by elevated levels of pyrazine compounds, benzyl nitrile, and methyl salicylate, which have been associated with nutty, almond-like, and minty aroma characteristics, respectively. Meanwhile, TD-inoculated batches showed higher relative abundances of methylpyrazine and 3-methylbutanoic acid, which are associated with nutty- and cheesy-associated aroma characteristics, respectively.

Although VOC profiles at 240 days differed markedly from those observed at 60 days, ganjang batches inoculated with *D. hansenii* (TD and TDWC) and/or *Wi. versatilis* (TC and TDWC) consistently exhibited significantly higher relative abundances of multiple VOCs (Fig. 8B). In contrast to the 60-day batches, TD-inoculated batches at 240 days showed significantly higher relative abundances of a broader range of VOCs, including methyl octanoate and 3-methyl-1-butanol (fruity-associated), 2-phenylpropenal (floral-associated), methyl tetradecanoate (fatty-associated), 3-methylbutanoic acid (cheesy-associated), guaiacol (smoky-associated), and methylpyrazine (nutty-associated). In contrast, TC-inoculated batches exhibited elevated levels of a more limited and distinct set of VOCs compared with those at 60 days, including 4-ethylguaiacol (smoky-associated), 4-ethylphenol (woody-associated), and the nutty compounds 2,5-dimethylpyrazine and 2,4,6-trimethylpyridine. TDWC-inoculated batches were enriched in methyl benzoic acid and benzaldehyde (almond-like-associated), tetramethylpyrazine (nutty-associated), and 3-octanol (mushroom-like-associated). Notably, even the yeast-free T-inoculated batches showed relatively higher abundances of fruity ester-associated compounds (methyl tridecanoate and methyl decanoate) at 240 days.

## 4 Discussion

Traditional ganjang in Korea is produced by soaking spontaneously fermented doenjang-meju without the use of defined starter cultures. Previous studies, including our own, have consistently detected diverse yeast taxa during ganjang fermentation and suggested that yeasts such as *Debaryomyces* and *Wickerhamomyces* contribute to metabolite and VOC production (Chun et al., 2021; Han et al., 2020; Jeong et al., 2022; Song et al., 2021). However, because traditional ganjang relies on spontaneous fermentation, the specific functional roles of individual yeasts have remained difficult to resolve. To address this limitation, we inoculated representative ganjang-associated yeasts in combination with *T. halophilus*, a key bacterial species in ganjang fermentation, and systematically evaluated their effects on pH dynamics, microbial communities, metabolites, and VOC profiles.

A central finding of this study is that yeast inoculation had little effect on overall microbial community structure (Fig. 2, Fig. 3) and primary metabolite composition (Fig. 4, Fig. 5) but exerted strong, fermentation stage-dependent effects on VOC profiles (Fig. 6, Fig. 7). Changes in pH, a key indicator of ganjang fermentation, closely tracked lactate accumulation during early fermentation (Fig. 1), consistent with the dominant role of *T. halophilus* as the primary lactic acid producer (Fig. 2). After removal of doenjang-meju, free sugars and amino acids declined, accompanied by a reduction in *Tetragenococcus* abundance. At later stages, *Debaryomyces*, a yeast previously reported to utilize lactate, became the predominant fungal taxon, suggesting that the pH shift observed after doenjang-meju removal may be associated with changes in microbial metabolism, including the activities of lactate-producing bacteria and lactate-utilizing microorganisms. Overall, the pH profiles, characterized by a rapid initial decrease followed by a gradual increase after doenjang-meju removal, were consistent with previous reports (Chun et al., 2021; Han et al., 2020; Jung et al., 2015). Nevertheless, the overall pH profiles were highly similar across all batches regardless of yeast inoculation, suggesting that yeast inoculation may cause only minor changes in ganjang fermentation characteristics.

Although absolute quantification provides improved ecological resolution for interpreting microbial dynamics in batch fermentation systems, it remains subject to several methodological limitations. Differences in cell lysis and DNA extraction efficiencies among microorganisms, particularly between taxa with distinct cell wall properties, may affect recovery rates during sample processing. In addition, PCR amplification and sequencing biases can introduce variation in read abundance among taxa because primer-binding affinity and amplification efficiency differ across marker gene sequences. Furthermore, variation in marker gene copy numbers among microorganisms may influence the estimation of absolute abundances at the taxon level. Therefore, the calculated values should be interpreted as estimated absolute gene copy abundances rather than direct measurements of viable cell numbers or exact microbial population sizes. Despite these methodological limitations, the spike-in normalization approach improved the resolution of temporal microbial dynamics across ganjang fermentation batches. In addition, microbial pellets from triplicate batches were pooled prior to sequencing, limiting the evaluation of biological variability among replicate fermentations. Therefore, differences in microbial community composition among treatments should be interpreted with appropriate caution. Absolute analysis clearly demonstrated that *T. halophilus* dominated the bacterial community throughout fermentation, confirming its central role in ganjang fermentation (Fig. 2). However, because sequencing was performed on pooled biological replicates, community differences should be interpreted as overall trends. In contrast, both inoculated yeasts and doenjang-meju-derived *Aspergillus* were present at very low absolute abundances during early fermentation, and *Debaryomyces* became abundant only after doenjang-meju removal (Fig. 3). The rapid decrease in pH coinciding with *T. halophilus* dominance may have contributed to limited early yeast proliferation, thereby limiting their contribution to overall community structure and primary metabolite dynamics.

Importantly, absolute analyses further revealed that reliance on relative abundance may overestimate the apparent ecological prominence of taxa such as *Bacillus* and *Aspergillus* when absolute abundance is not considered. Although these taxa showed high relative abundances during the early stage, absolute analyses demonstrated that they remained at very low levels throughout fermentation (Fig. 2, Fig. 3). This finding indicates that they were not actively proliferating during fermentation but were instead passively carried over from the doenjang-meju bricks, and that their apparent “decline” reflected their initial rarity rather than true disappearance. Overall, this study demonstrates that reliance on relative abundance alone substantially overestimates the contributions of *Bacillus*, *Aspergillus*, and several other microbes during fermentation. Therefore, absolute quantification is essential for accurately identifying dominant bacterial and fungal contributors and for characterizing microbial dynamics and fermentation features in batch fermentations such as ganjang (Chun et al., 2021).

Although yeasts remained at low absolute abundance during early fermentation, their limited abundance does not preclude functional significance. VOCs are generally produced in much smaller quantities than primary metabolites and are widely recognized as compounds associated with aroma-related characteristics in fermented foods. This finding highlights that, in ganjang fermentation, VOC-associated metabolic processes are governed by metabolic specialization rather than population size. Therefore, even modest yeast growth may contribute to differences in VOC profiles through the conversion of available precursors, such as amino acids, phenolic acids, and fatty acid derivatives, into VOCs. This ecological pattern suggests that yeasts may contribute primarily to VOC formation and secondary metabolism rather than as dominant drivers of bulk metabolic fluxes. Moreover, the consistently low absolute abundance of inoculated yeasts suggests that, in the absence of strong bacterial acidification by *T. halophilus* (i.e., without *T. halophilus* inoculation), yeasts might proliferate more extensively and potentially exert broader influences not only on VOC production but also on overall community structure and primary metabolic processes.

Doenjang-meju bricks are rich sources of free sugars and amino acids generated through enzymatic degradation of macromolecules by *Aspergillus* and *Bacillus* (Han, Baek, Choi and Jeon, 2024a, Han, Baek, Choi and Jeon, 2024b; Kim et al., 2022). Accordingly, removal of the doenjang-meju bricks resulted in marked declines in these metabolites at later fermentation stages. Flavonoid aglycone levels showed slight differences across batches, suggesting that certain yeast combinations may influence the liberation of aglycones from flavonoid glycosides during ganjang fermentation (Fig. 4D). However, the overall metabolite profiles remained highly similar across ganjang batches regardless of yeast inoculation (Fig. 4). This finding aligns with the observation that bacterial and fungal community structures also showed no major batch-specific differences, indicating that the inoculation of different yeast sets had minimal influence on the overall metabolite composition of ganjang. Consistent with this pattern, multivariate analyses showed no clear separation of metabolite profiles among batches at either 60 or 240 days, with only minor differences observed in a limited subset of amino acids and flavonoid aglycones (Fig. 5). PERMANOVA further confirmed that yeast inoculation had no significant effects on free sugars, organic acids, or amino acids. Collectively, these results indicate that variation in yeast composition exerts minimal influence on primary metabolite production during ganjang fermentation.

In contrast, VOC profiles exhibited pronounced batch-specific differences, indicating that although different yeast starters had limited effects on microbial community composition and primary metabolite production, they strongly influenced the VOC profiles of ganjang. Despite their low estimated absolute abundances, were associated with distinct VOC profiles (Fig. 6, Fig. 7). Among the tested yeasts, *D. hansenii* and *Wi. versatilis* showed the strongest associations with VOC differentiation, particularly during the late stage of ganjang fermentation (Fig. 8B). After removal of the doenjang-meju bricks, *Debaryomyces* became the predominant fungal genus in all batches, regardless of yeast inoculation. Before doenjang-meju removal, *D. hansenii* and *Wi. versatilis* were clearly detected only in batches inoculated with these yeasts (Fig. 3). Notably, these early differences coincided with higher diversity and abundance of VOCs in the corresponding ganjang batches, suggesting that VOC differentiation among batches was more evident prior to doenjang-meju removal, when carbohydrates and amino acids were most abundant (Fig. 4).

These observations are consistent with the documented VOC-producing capacities of *Wi. versatilis* and *D. hansenii*, particularly their phenolic acid decarboxylation activity and amino acid catabolism under high-salt conditions (Jeong et al., 2022; Qi et al., 2014). *Wi. versatilis* is known to possess phenolic acid decarboxylation activity, enabling the decarboxylation of hydroxycinnamic acids (ferulic acid and *p*-coumaric acid) via ferulic acid decarboxylase (FDC1), followed by reduction of vinyl derivatives to ethyl phenols via vinyl reductase (VRD1), potentially contributing to the formation of the tentatively identified VOCs 4-ethylguaiacol and 4-ethylphenol, respectively (Fig. S2A) (Hou et al., 2016; Suezawa & Suzuki, 2007). These reactions may contribute to smoky- and woody-associated aroma characteristics under high-salinity conditions. The elevated relative abundance of 4-ethylguaiacol in the TC batch is consistent with previous reports that *Wi. versatilis* can convert 4-vinylguaiacol to 4-ethylguaiacol (Fig. 7, Fig. 8). In addition, it may produce fruity esters and higher alcohols through the Ehrlich pathway coupled with alcohol acetyltransferase activity (Fig. S2B) (Wang, Zhen, et al., 2025; Yoo et al., 2025). Similarly, *D. hansenii* efficiently catabolizes amino acids and organic acids under high-salt conditions, producing branched-chain alcohols and acids (e.g., 3-methyl-1-butanol and 3-methylbutanoic acid), as well as precursors associated with nutty- and cheesy-associated aroma characteristics (Fig. S2C) (Jeong et al., 2022; Yoo et al., 2025). This interpretation is consistent with the elevated relative abundance of 3-methylbutanoic acid in the TD batch. Together, these metabolic characteristics provide a possible explanation for the observed differences in relative VOC abundances in ganjang batches inoculated with *D. hansenii* and *Wi. versatilis*.

In contrast, *Wo. anomalus* exhibited a limited impact on VOC formation in this study (Fig. 7), despite its reported contribution to the generation of key aroma compounds in soy sauce fermentation (Liu et al., 2025). Previous studies have shown that *Wo. anomalus* is generally detected at lower abundances than *D. hansenii* or *Wi. versatilis* in soy sauce fermentations (Han et al., 2020; Wang et al., 2022), suggesting that *Wo. anomalus* may be less well adapted to the high-salt and/or low-pH conditions characteristic of ganjang fermentation. This lower apparent persistence may partly explain the comparatively minor influence of *Wo. anomalus* inoculation on VOC formation compared with *D. hansenii* and *Wi. versatilis* during ganjang fermentation.

The effects of yeast inoculation were strongly dependent on fermentation stage. *Wi. versatilis* inoculation was associated with higher relative abundances of smoky-, woody-, fruity-, and fatty-associated VOCs during mid-fermentation (60 days), whereas *D. hansenii* was associated with higher relative abundances of nutty-, cheesy-, and floral-associated VOCs during late fermentation (240 days) (Fig. 8). VOC differentiation among batches was more pronounced at 60 days than at 240 days, reflecting stronger starter-driven effects during early fermentation. These findings raise the possibility that stage-specific or sequential yeast inoculation strategies may influence VOC profile development during ganjang fermentation. However, this hypothesis requires direct validation because all treatments in the present study included *T. halophilus* as a common background starter. One possible explanation is that early acidification by *T. halophilus* may have limited yeast proliferation during the initial fermentation stage. Therefore, delayed yeast inoculation could potentially alter yeast activity and VOC profiles, although this possibility remains speculative and requires experimental validation. This observation raises the possibility that yeast inoculation after doenjang-meju removal may result in different VOC profiles; however, such effects were not directly tested in this study.

Nevertheless, yeast-associated differences persisted into late fermentation, indicating that early microbial activity may be associated with long-lasting differences in VOC profiles. In particular, the enrichment of smoky phenols (e.g., 4-ethylguaiacol), nutty pyrazines, and fruity esters in specific inoculation sets during late fermentation indicates that targeted yeast selection may enable controlled modulation of aroma-associated VOC profiles. These discriminant VOCs therefore provide practical markers for designing starter combinations tailored to desired ganjang VOC profiles. Additionally, the accumulation of certain esters even in yeast-free batches suggests that prolonged fermentation can also contribute to VOC formation, including ester production, through bacterial metabolism and/or non-enzymatic reactions.

Because VOCs were analyzed as normalized relative abundances rather than absolute concentrations, the present data should be interpreted as comparative, semi-quantitative differences among treatments rather than direct measures of aroma intensity or sensory impact. Finally, because all treatments included *T. halophilus* as a common background starter, the present experimental design does not allow direct evaluation of yeast effects in the absence of bacterial acidification or comparison with a no-*T. halophilus* control. Consequently, the proposed benefits of delayed or sequential yeast inoculation should be regarded as hypotheses generated from the observed associations rather than validated fermentation strategies. Future studies incorporating treatments without *T. halophilus* and varying yeast inoculation timings will be required to test these possibilities directly.

## 5 Conclusion

This study demonstrates that yeast inoculation selectively shapes VOC profiles during ganjang fermentation, while having minimal influence on overall microbial community structure and primary metabolite composition. These findings indicate that microbial composition and nonvolatile metabolites alone cannot fully explain VOC-associated aroma characteristics in ganjang. Rather, VOC formation is highly stage-dependent and markedly more responsive to yeast inoculation than bulk metabolic profiles. In particular, inoculation with *D. hansenii* and/or *Wi. versatilis* was associated with distinct VOC profiles characterized by differences in the relative abundances of discriminant VOCs across fermentation stages. Notably, yeasts present at low estimated absolute gene copy abundances were associated with pronounced changes in VOC profiles, suggesting that changes in VOC profiles are not necessarily proportional to microbial abundance. Collectively, these results provide insights that may support the rational design of yeast starters to modulate VOC composition and diversity, thereby contributing to differences in VOC profiles in ganjang. More broadly, this study highlights the value of integrating absolute microbial quantification with multi-omics approaches to enable evidence-based starter design in complex fermentation environments. From an industrial perspective, the findings provide a basis for investigating whether stage-specific selection and timing of yeast starter application can be used to modulate aroma-associated VOC profiles in ganjang fermentation. Further studies are needed to validate this possibility under controlled fermentation conditions.

## CRediT authorship contribution statement

**Dong Min Han:** Writing – original draft, Visualization, Methodology, Formal analysis. **Ju Hye Baek:** Methodology, Investigation. **Dae Gyu Choi:** Investigation. **Jae Kyeong Lee:** Formal analysis. **Byung Hee Chun:** Supervision, Investigation. **Che Ok Jeon:** Writing – review & editing, Supervision, Funding acquisition, Conceptualization.

## Declaration of competing interest

The authors declare that they have no known competing financial interests or personal relationships that could have appeared to influence the work reported in this paper.

## Data Availability

The bacterial 16S rRNA gene and fungal ITS2 region sequencing data generated in this study have been deposited in the NCBI Short Read Archive (SRA) under accession numbers SRR36038459–SRR36038498 and SRR36153464–SRR36153503, respectively, as part of BioProject PRJNA1365049.
