# Supplementary material for: Effects of yeast inoculation on community, metabolites, and flavors in ganjang, a traditional Korean fermented soy sauce
Source: Food Chem X. 2026 Jul 13;37:104200. doi: 10.1016/j.fochx.2026.104200 (PMC13382134; doi:10.1016/j.fochx.2026.104200)
Supplement: Supplementary file 1 — Supplementary material 1 [file mmc1.docx]

**Effects of yeast inoculation on community, metabolites, and flavors in ganjang, a traditional Korean fermented soy sauce**

Dong Min Han^1,†^, Ju Hye Baek^1,†^, Dae Gyu Choi^1^, Jae Kyeong Lee^1^, Byung Hee Chun^2,^*, and Che Ok Jeon^1,^*

^1^*Department of Life Science*, *Chung-Ang University*, *Seoul 06974*, *Republic of Korea*

^2^*Department of Microbiology, Pukyong National University, Busan 48513, Republic of Korea*

* Corresponding author (Che Ok Jeon)

^†^ These authors contributed equally to this study.

**Fig. S1**. Schematic overview of ganjang preparation with different inoculation sets from fermented doenjang-meju bricks, the fermentation process, and ganjang sampling for subsequent analyses.


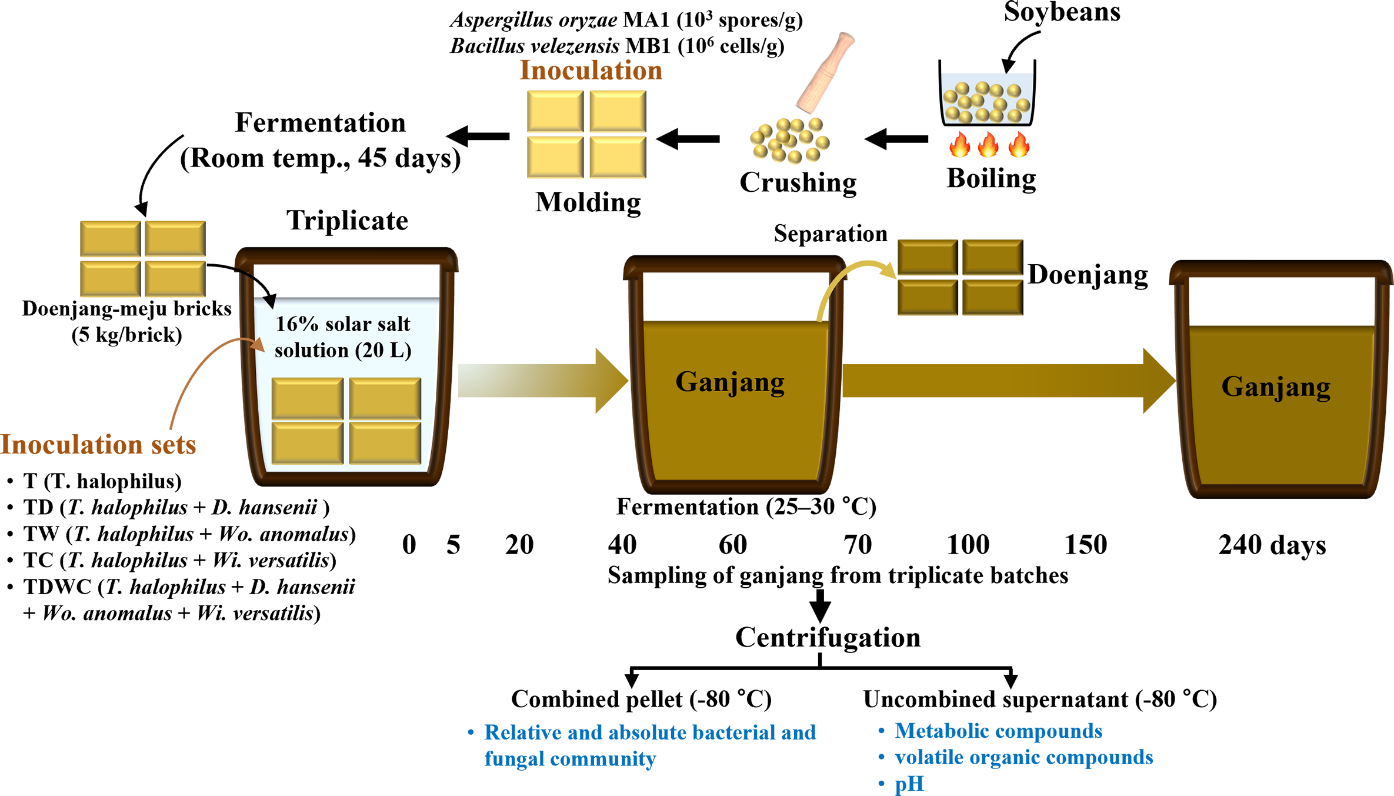


**Fig. S2.** Proposed phenolic acid decarboxylation pathway for the production of 4-ethylguaiacol and 4-ethylphenol by *Wickerhamiella versatilis* (A), the Ehrlich and ester synthesis pathways for the production of higher alcohols and esters by *Wi. versatilis* (B), and pathways for the production of branched-chain alcohols and acids (e.g., 3-methyl-1-butanol and 3-methylbutanoic acid) by *Debaryomyces hansenii* (C) during ganjang fermentation.

**
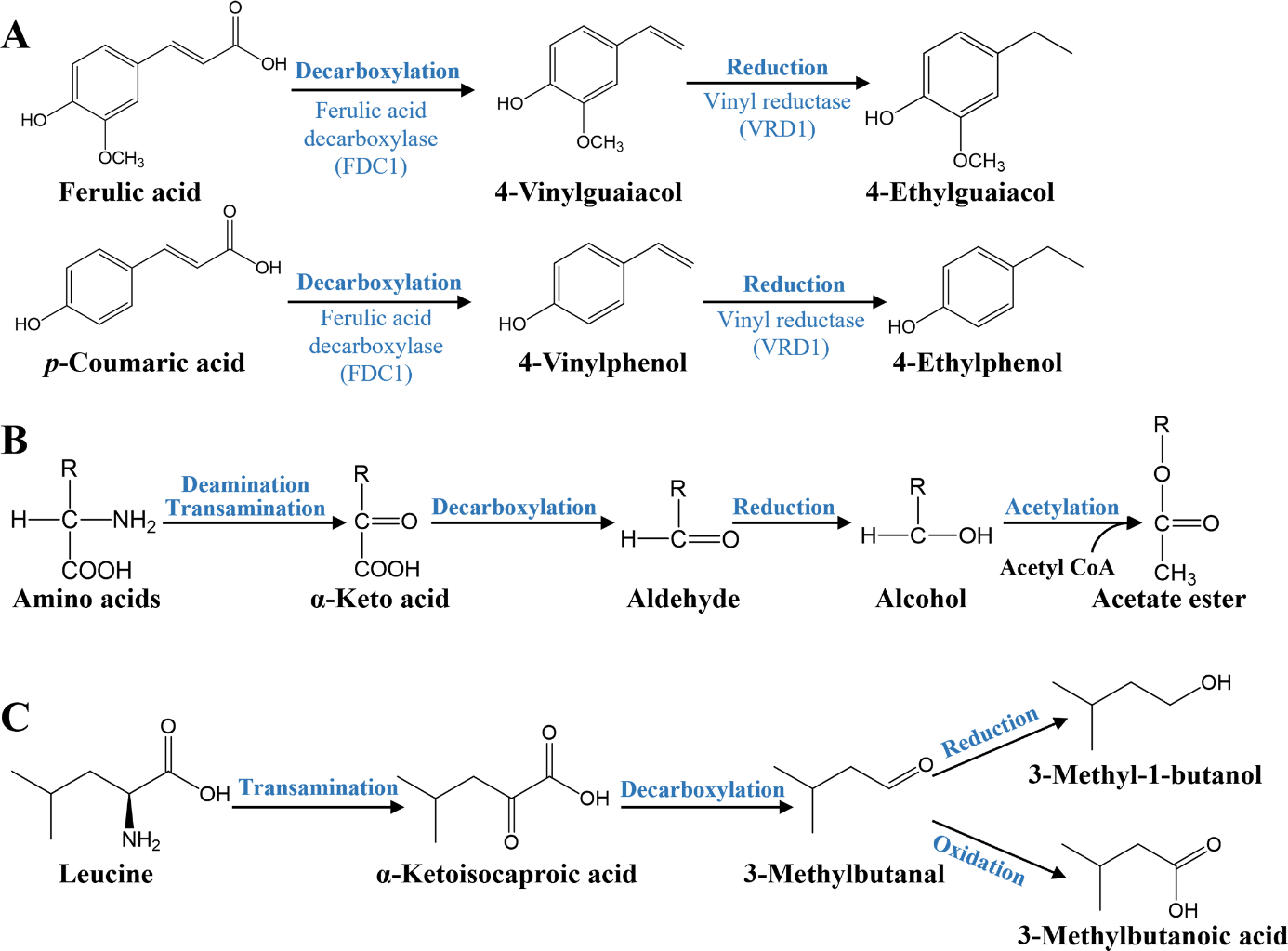
**

**Table S1**. Volatile organic compounds identified from ganjang batches inoculated with *Tetragenococcus halophilus* , *T. halophilus* and *Debaryomyces hansenii*, *T. halophilus* and *Wickerhamomyces anomalus*, *T. halophilus* and *Wickerhamiella versatilis*, and *T. halophilus*, *D. hansenii*, *Wo. anomalus*, and *Wi. versatilis*. Detailed data are provided in a separate Excel file (supplementary Table S1).

**Table S2.** Results of ANOVA based on profiles of volatile organic compounds identified from 60 and 240 days of ganjang batches inoculated with *Tetragenococcus halophilus*, *T. halophilus* and *Debaryomyces hansenii*, *T. halophilus* and *Wickerhamomyces anomalus*, *T. halophilus* and *Wickerhamiella versatilis*, and *T. halophilus*, *D. hansenii*, *Wo. anomalus*, and *Wi. versatilis.* Detailed data are provided in a separate Excel file (supplementary Table S2).
